# Moderately Elevated Temperature Offsets the Adverse Effects of Waterlogging Stress on Tomato

**DOI:** 10.3390/plants13141924

**Published:** 2024-07-12

**Authors:** Junqin Wen, Shumei Sui, Jie Tian, Yanhai Ji, Zhen Wu, Fangling Jiang, Carl-Otto Ottosen, Qiwen Zhong, Rong Zhou

**Affiliations:** 1 Key Laboratory of Qinghai-Tibetan Plateau Biotechnology of Ministry of Education, Academy of Agriculture and Forestry Sciences of Qinghai University (Qinghai Academy of Agriculture and Forestry Sciences), Xining 810016, China; 2021990055@qhu.edu.cn (J.W.);; 2 National Engineering Research Center for Vegetables, Beijing Vegetable Research Center, Beijing Academy of Agriculture and Forestry Science, Beijing 100097, China; 3 College of Horticulture, Nanjing Agricultural University, Nanjing 210095, China; zpzx@njau.edu.cn (Z.W.); jfl@njau.edu.cn (F.J.); 4 Department of Food Science, Aarhus University, Agro Food Park 48, DK-8200 Aarhus, Denmark; coo@food.au.dk

**Keywords:** tomato, temperature, waterlogging, gas exchange, water-use efficiency

## Abstract

Global warming and waterlogging stress due to climate change are expected to continue influencing agricultural production worldwide. In the field, two or more environmental stresses usually happen simultaneously, inducing more complex responses in plants compared with individual stresses. Our aim was to clarify how the two key factors (temperature and water) interacted and influenced physiological response and plant growth in tomatoes under ambient temperature, moderately elevated temperature, waterlogging stress, and moderately elevated temperature and waterlogging stress. The results showed that leaf photosynthesis was inhibited by waterlogging stress but enhanced by elevated temperature, as shown by both the light- and temperature-response curves. The elevated temperature decreased leaf water-use efficiency, but enhanced plant growth and fresh and dry weights of plants under both normal water supply and waterlogging stress conditions. Elevated temperature generally decreased the anthocyanin and flavonol index in tomato leaves compared with the control temperature, regardless of water status. The increase in the optimal temperature was more pronounced in plants under normal irrigation than under waterlogging stress. Waterlogging stress significantly inhibited the root length, and leaf number and area, while the moderately elevated temperature significantly enhanced the leaf number and area. Overall, the moderately elevated temperature offset the effects of waterlogging stress on tomato plants, as shown by leaf gas exchange, plant size, and dry matter accumulation. Our study will improve the understanding of how tomatoes respond to increasing temperature and excess water.

## 1. Introduction

Climate change affects the climate worldwide, both with extreme temperatures and massive precipitation [1]. Global warming increased by 0.87 °C from 2006 to 2015 and is likely to increase by 1.5 °C from 2030 to 2052 compared with pre-industrial levels [1,2]. More importantly, the environmental changes, such as global warming and waterlogging stress, induced by climate change will continue affecting whole ecosystems and agricultural production systems worldwide [2].

Global climate change has resulted in a higher frequency of extreme weather events, including heat waves, reducing plant growth and yield [3]. We previously found that moderately and acutely elevated temperatures can induce distinct responses from the perspective of miRNAs and their target genes [4]. In most cases and regions, global warming causes moderately elevated temperatures.

With the accelerated pace of climate change, both the frequency and intensity of heavy rainfall or flooding events are expected to increase, resulting in waterlogging stress on agricultural production [5,6,7]. Waterlogging stress appears when the root zone of plants is flooded due to excessive water in the soil or growing media [8]. When waterlogging occurs, air is expelled from the soil pores and plant roots are quickly exposed to an anaerobic environment due to the low diffusion rate of oxygen in water, which is detrimental to the plant [9,10]. On the other hand, waterlogging stress can induce leaf stomata closure, chlorophyll degradation, and senescence of plants, which ultimately leads to decreased photosynthetic rate and low yield in plants [11,12].

Due to global warming, waterlogging stress and elevated temperature can occur simultaneously and have negative impacts on agricultural production. However, currently, most studies still focus on the effects of individual factors on plants, e.g., high temperature [3,13] or waterlogging [10,14,15]. To improve understanding of plant responses to changing climate, and to increase their resilience, there is an urgent need to study how these two key environmental factors interact and influence the physiology of plants.

The photosynthetic apparatus in the chloroplast is one of the components that is sensitive to high temperature [13] and excessive water [14,16]. The optimal day/night temperatures for the growth of tomatoes are 25~30/20 °C [17], and heat-sensitive tomato cultivars exhibited swollen chloroplasts containing decomposed starch grains after seven days of heat stress (36/28 °C) [13]. We previously found that, after 8 days of waterlogging stress, the chloroplasts of tomatoes were damaged, as indicated by partially disintegrated grainy lamellae and impaired lamellar structure [16]. Photosynthesis in tomatoes was negatively affected by heat stress [13] and waterlogging [16]. Moreover, the negative effects of waterlogging in tomatoes included not only decreased leaf photosynthetic capacity, but also low flower and fruit set, especially when the stress occurred at the reproductive stage [15]. Waterlogging can become one of the biggest challenges for tomato production in the field, particularly due to more irregular rain patterns. Lin et al. (2016) found that the cultivar ‘L6138’ (*Solanum peruvianum*) showed the best performance among 44 wild tomato lines that had been waterlogged at +38 °C for 48 h, as indicated by the highest ASA, shoot growth, chlorophyll content, and chlorophyll fluorescence parameters [17]. Differentially expressed proteins play roles in protein structure maintenance, metabolism, and photosynthesis in tomatoes that had been waterlogged at +38 °C for 72 h [17].

This study aimed to clarify the responses of leaf photosynthesis and metabolites, as well as plant dry-matter accumulation, in tomato plants under control, moderately elevated temperature, or waterlogging stress conditions, or under a combination of these conditions. Our hypothesis was that the moderately elevated temperature could alleviate the negative effects of waterlogging stress on tomatoes. This study will help us to understand the physiological responses to waterlogging stress accompanied by increasing temperature, and to potentially increase the resilience of tomatoes to a changing climate.

## 2. Results

**Figure 1:**
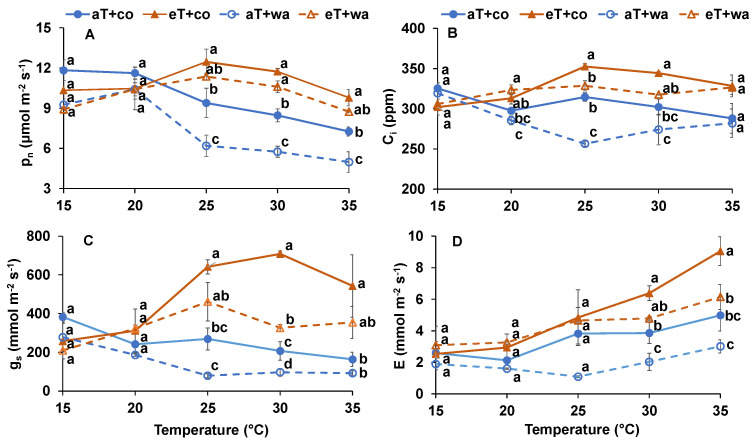
Temperature-response curve including (**A**) P_n_, (**B**) C_i_, (**C**) g_s_, and (**D**) E of tomato plants grown under four treatments. aT + co, eT + co, aT + wa, and eT + wa indicate 20 °C + controlled irrigation, 30 °C + controlled irrigation, 20 °C + waterlogging, and 30 °C + waterlogging, respectively. The data shown are means of three replicates ± SEM. Different letters above the lines indicate significant differences between the treatments at the same measured temperature (ANOVA, *p* < 0.05).

The P_n_ (net photosynthetic rate) was significantly lower at aT + wa than aT + co when the measured temperatures were 25 °C, 30 °C, and 35 °C (Figure 1A), indicating that waterlogging stress reduced the photosynthetic capacity. By comparison, a moderately elevated growth temperature (30 °C) enhanced the P_n_, as it was lower at 20 °C than 30 °C when the measured temperatures were 25 °C, 30 °C, and 35 °C (Figure 1A). At temperatures of 25 °C and 30 °C, the C_i_ (intracellular CO_2_ concentration) and g_s_ (stomatal conductance) at eT + co and eT + wa significantly increased compared with aT + co and aT + wa, respectively (Figure 1B,C). Waterlogging significantly decreased C_i_ at 25 °C and g_s_ at 30 °C (Figure 1B,C). At the high temperatures (30 °C and 35 °C), the E (transpiration rate) at eT + co and eT + wa significantly increased compared with aT + co and aT + wa (Figure 1D).

**Figure 2:**
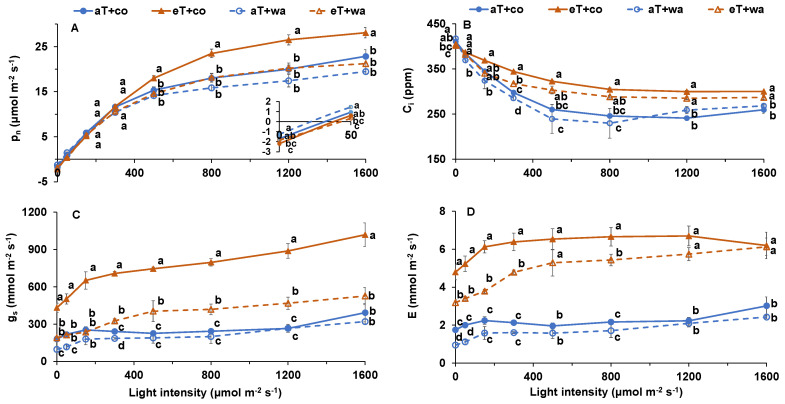
Light-response curve included (**A**) P_n_, (**B**) C_i_, (**C**) g_s_, and (**D**) E of tomato plants grown under four treatments. aT + co, eT + co, aT + wa, and eT + wa indicate 20 °C + controlled irrigation, 30 °C + controlled irrigation, 20 °C + waterlogging, and 30 °C + waterlogging, respectively. The data shown are means of three replicates ± SEM. Different letters above the lines indicate significant differences between the treatments at the same measured temperature (ANOVA, *p* < 0.05).

**Figure 3:**
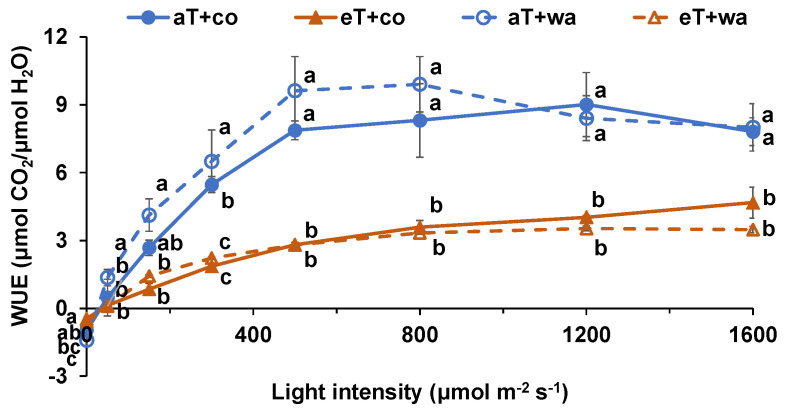
Water-use efficiency (WUE) of tomato plants grown under four treatments. aT + co, eT + co, aT + wa, and eT + wa indicate 20 °C + controlled irrigation, 30 °C + controlled irrigation, 20 °C + waterlogging, and 30 °C + waterlogging, respectively. The data shown are means of three replicates ± SEM. Different letters above the lines indicate significant differences between the treatments at the same measured temperature (ANOVA, *p* < 0.05).

The P_n_ was significantly lower at aT + co, aT + wa, and eT + wa compared with eT + co when the light level was ≥500 μmol m^−2^ s^−1^ (Figure 2A). The Ci at aT + co and aT + wa was significantly lower compared with eT + co, at light ≥ 300 μmol m^−2^ s^−1^ (Figure 2B). The eT + co showed the highest gs in all cases (Figure 2C). The E at 30 °C was significantly higher than 20 °C regardless of waterlogging treatment (Figure 2D). The WUE (water-use efficiency) of plants at 20 °C was significantly increased compared with 30 °C regardless of waterlogging treatment when the light level was ≥300 μmol m^−2^ s^−1^ (Figure 3). The T_opt_ (optimum temperature) of plants at eT was significantly higher than at aT regardless of waterlogging (Table 1). The Rd (dark respiration) was significantly lowest at aT + wa, while the Φ (apparent quantum yield) of plants at aT + co was higher than that at eT + co and eT + wa (Table 1). The LCP (light compensation point) at eT + co and eT + wa was higher than that at aT + co and aT + wa (Table 1). Moreover, the A_max_ (maximum net assimilation rate) at eT + co was higher than at aT + wa and eT + wa (Table 1).

**Figure 4:**
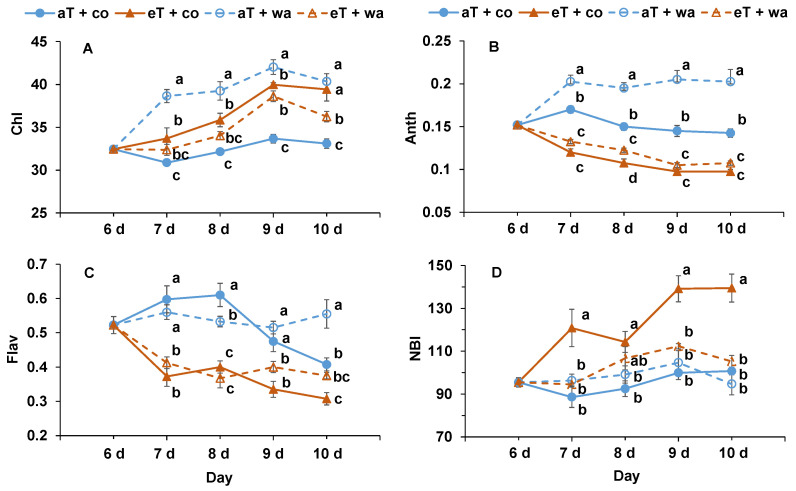
(**A**) Chl (chlorophyll index), (**B**) Anth (anthocyanin index), (**C**) Flav (flavonol index), and (**D**) NBI (nitrogen balanced index) of tomato plants grown under four treatments. aT + co, eT + co, aT + wa, and eT + wa indicate 20 °C + controlled irrigation, 30 °C + controlled irrigation, 20 °C + waterlogging, and 30 °C + waterlogging, respectively. The data shown are means of three replicates ± SEM. Different letters above the lines indicate significant differences between the treatments at the same measured temperature (ANOVA, *p* < 0.05).

The Chl (chlorophyll index) of the leaves was the highest at aT + wa, followed by eT + co and aT + co from day 7 to day 10 (Figure 4A). The Anth (anthocyanin index) was highest at aT + wa, followed by aT + co, and lowest at eT + co and eT + wa from day 7 to day 10 (Figure 4B). The Flav (flavonol index) was highest at aT + co from day 7 to day 9, and was significantly higher than that at eT + co and eT + wa (Figure 4C). The NBI (nitrogen balanced index) of plants at eT + co was significantly higher than at aT + co or aT + wa from day 7 and day 10 (Figure 4D).

**Figure 5:**
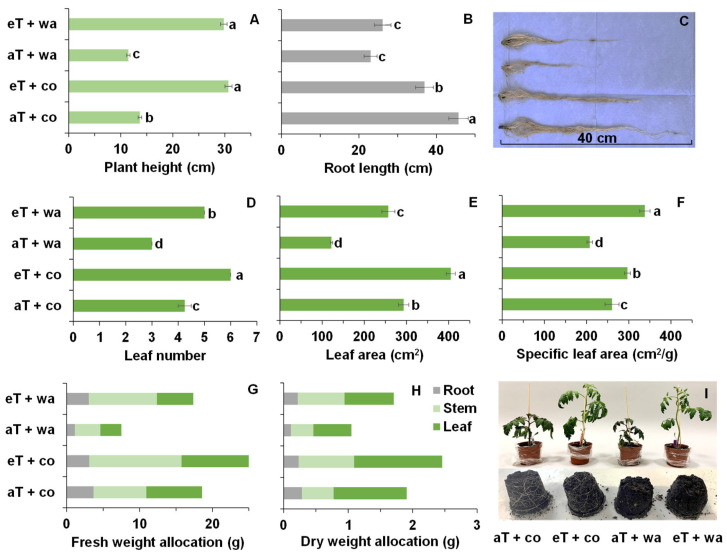
(**A**) Plant height, (**B**) root length, (**C**) root photo, (**D**) leaf number, (**E**) leaf area, (**F**) specific leaf area, (**G**) fresh weight allocation, (**H**) dry weight allocation, and (**I**) plant photo of tomato plants grown under four treatments. aT + co, eT + co, aT + wa, and eT + wa indicate 20 °C + controlled irrigation, 30 °C + controlled irrigation, 20 °C + waterlogging, and 30 °C + waterlogging, respectively. The data shown are means of three replicates ± SEM. In (**G**,**H**), only the mean values are shown to compare the fresh and dry weight allocation of root, stem, and leaf. Different letters indicate significant differences between the treatments (ANOVA, *p* < 0.05).

Plant height was significantly higher at moderately elevated temperatures regardless of water treatment (Figure 5A). The root length of the control plants was significantly longer than that of the waterlogged plants (Figure 5B,C). The leaf number of plants grown at 30 °C was higher than that at 20 °C, but the waterlogged plants had fewer leaves than the control plants (Figure 5D). The leaf area of control plants was significantly higher than that of waterlogged plants, but, in general, higher temperature resulted in larger leaf areas (Figure 5E). The plants at eT + wa and aT + wa showed the highest and lowest specific leaf area (Figure 5F). The fresh and dry weights of plants at eT + co were the highest, followed by eT + wa and aT + co, while those at aT + wa was the lowest (Figure 5G,H). The plants grown at 30 °C/26 °C were taller than those grown at 20 °C/16 °C regardless of waterlogging treatment, but waterlogging generally reduced height (Figure 5I).

## 3. Discussion

Under natural conditions, multiple abiotic stresses usually co-occur, which can cause severe damage to plants [16,18,19]. It is notable that combined stresses can induce unique responses in plants, which can differ from the responses caused by the individual factors [19,20]. Therefore, identifying the interactive and complex physiological effects of moderately elevated temperatures and waterlogging stress on plants will provide new understanding of combined stress effects.

It is well known that waterlogging stress can severely inhibit tomato photosynthesis, growth, and development [10,16,19,21]. Here, waterlogging significantly decreased the P_n_ at the ambient growth temperature when the measured temperature was ≥25 °C (Figure 1A). The reason for the negative effect of waterlogging on P_n_ could be reduced gas diffusion, oxygen shortage for respiration, or decreased ATP production to support cellular biological processes [19,22,23]. Moreover, waterlogging decreased the g_s_ and E (Figure 2C,D), as seen in previous studies [24].

However, the responses differed at the higher temperature, where the g_s_ and E were higher compared with the control, regardless of water status (Figure 1 and Figure 2). By comparison, moderately increased temperature increased the P_n_ due to higher g_s_, E, and C_i_ when the measured temperature was ≥25 °C, regardless of water supply (Figure 1). Moreover, P_n_ was higher at elevated temperature and controlled irrigation than ambient temperature and controlled irrigation (Figure 2A). The temperature- and light-response curves showed the positive effects of the moderately elevated temperature on leaf photosynthetic capacity. Short-term moderate heat stress (35/18 °C day/night) did not significantly decrease the P_n_ of pea (*Pisum sativum* L.) when analyzing both light- and temperature-response curves [25]. The elevated temperature did not always reduce P_n_. The reason could have been that moderately higher air temperatures may enhance plant nitrogen content, which is primarily related to photosynthetic capacity, since the proteins of the Calvin–Bensen cycle and thylakoids contain most of the leaf nitrogen [26].

Apart from the photosynthesis, positive effects of moderately elevated temperature on plant growth and dry matter accumulation were observed. The plants grown at elevated temperature had increased height, higher leaf numbers, and larger leaf area and specific leaf area, as well as increased fresh and dry weights, compared with plants grown at the control temperature, not only under normal irrigation, but also under waterlogging (Figure 5). However, it is notable that water-use efficiency was lower at elevated temperatures than in the control at the most of light intensities, regardless of irrigation, even though the positive effects of moderately elevated temperature were seen (Figure 3).

Leaf optimal temperature increased by 9.7 °C and 4.5 °C, respectively, at normal irrigation and waterlogging, when the tomato plants were grown at the moderated elevated temperature, as compared with the control (Table 1). Growth temperature altered the temperature dependence of photosynthetic rate, reflecting a homeostatic response to sustaining the photosynthetic rate at the growth temperature [27]. With increasing growth temperature, the optimal temperature increased the P_n_ in many species [27,28]. Here, the optimal temperature of tomato plants increased when the growth temperature increased; however, the waterlogging stress reduced the increase in the optimal temperature. Therefore, the shift in the optimal temperature was more pronounced in normally irrigated plants as compared with waterlogged plants. Altogether, we concluded that moderately elevated temperatures offset the negative effects of waterlogging stress on tomato plants, as indicated by leaf gas exchange, plant growth, and dry matter accumulation.

## 4. Materials and Methods

### 4.1. Plant Growth and Experimental Design

The tomato cultivar ‘Qianxi’ with indeterminate growth type and red cherry fruit (Known-you seed Co., Ltd., Taiwan, China) were used as plant material in the current study. The seeds were sown in a greenhouse (Aarhus University, Aarhus, Denmark) at 24 °C and approximately 60% relative humidity (RH). The greenhouse had LED lamps (FL300, Senmatic) which ensured that the light intensity was kept above 300 µmol m^−2^ s^−1^ photosynthetic photon flux density (PPFD) during daytime. Nutrient solution with 2.4 mS/cm EC, 180 ppm N, 34 ppm P, and 259 ppm K was applied three weeks after sowing. The 20-day-old tomato seedlings were transferred to the climate chambers to acclimate for two days. The daytime (7:00 a.m.–21:00 p.m.), with 300 µmol m^−2^ s^−1^, and the nighttime (21:00 p.m.–7:00 a.m.) were 14 h/10 h. The temperature was 20 °C/16 °C (day/night) with 60% RH during acclimation. The 22-day-old tomato seedlings were, after acclimation, grown under four treatments including (1) 20 °C/16 °C and controlled normal irrigation as ‘aT + Co’, (2) 30 °C/26 °C and controlled normal irrigation as ‘eT + Co’, (3) 20 °C/16 °C and waterlogging stress as ‘aT + Wa’, and (4) 30 °C/26 °C and waterlogging stress as ‘eT + Wa’.

During the treatments, the waterlogging stress was applied as described in our previous study [19], where the pots with the seedlings were submerged in water. This was achieved by putting each pot with seedlings in another, bigger pot filled with water. There were 20 plants per treatment. The treatments lasted for 10 days (day 0–10). The plants under all treatments were manually irrigated with 100 mL nutrient solution per pot on days 3, 6, and 9. The first fully expanded leaf was selected for all the measurements below, with three replicates.

### 4.2. Gas Exchange Measurements

Temperature and light responses were measured on days 8 and 9 using a portable photosynthesis system (CIRAS-2, PP Systems, Amesbury, MA, USA). The gas-exchange parameters, including P_n_, C_i_, g_s_, and E, were recorded every 10 s until P_n_ and g_s_ were steady. The average value of the last minute was used for the analysis. It is notable that the plants were grown in the same conditions during the treatments and only the changes in temperature and light levels were achieved by changing the settings of the leaf cuvette, but not the whole range of growth conditions of the plants.

For the temperature-response curve, the light intensity of the leaf cuvette was set to 300 µmol m^−2^ s^−1^. The temperature ranged from 15 °C to 35 °C with 5 °C intervals. The T_opt_ was calculated as the vertex of a hyperbola fitted for the temperature-response curve.

For the light-response curve, the temperature setting of the leaf cuvette was 20 °C and 30 °C for the aT and eT treatments, respectively. The light intensity started from a PPFD of 300 followed by 500, 800, 1200, 1600, 300, 150, 50, and 0 μmol m^−2^ s^−1^. Leaf water-use efficiency (WUE = P_n_/E) under different light intensities was calculated based on the results from the light-response curve. The R_d_, apparent quantum yield (Φ), LCP, and A_max_ were calculated by curve fits for the light-response curve using Response Curve Fitting 1.0 [29].

### 4.3. Metabolite Measurements

Plant metabolite parameters, including NBI, Chl, Flav, and Anth, were non-destructively measured on days 6 to 10 using Dualex (FORCE-A, Orsay, France).

### 4.4. Plant Harvest

On day 10, plant height was measured using a ruler and leaf numbers were counted. Afterwards, plants were destructively harvested, and the fresh weight of roots, stems, and leaves was measured. Leaf area was measured using leaf area meter (model 3100, LICOR, Lincoln, NE, USA). Length of root was investigated using a ruler after carefully washing the root. Dry weights of roots, stems, and leaves were investigated after putting the different sections in the oven at 85 °C for 48 h. Specific leaf area (cm^2^/g) was calculated as the ratio between leaf area and leaf dry weight.

### 4.5. Data Analysis

The data were analyzed using SPSS 16.0 (SPSS Inc., Chicago, IL, USA). The ANOVA with Tukey’s post hoc test was performed, with a significance level at *p* ≤ 0.05.

## Figures and Tables

**Table 1:** Optimal temperature (T_opt_), dark respiration (Rd), apparent quantum yield (Φ), light compensation point (LCP), and maximum net assimilation rate (A_max_) of tomato plants grown under four treatments.

Group	T_opt_ (°C)	Rd/ (μmol m^−2^ s^−1^)	Φ/ (mol/mol)	LCP/ (μmol m^−2^ s^−1^)	A_max_/ (μmol m^−2^ s^−1^)
aT + co	16.7 ± 1.69 b	1.89 ± 0.064 a	0.066 ± 0.0053 a	30.2 ± 1.42 b	28.8 ± 2.59 ab
eT + co	26.4 ± 1.27 a	2.19 ± 0.214 a	0.054 ± 0.0017 b	41.2 ± 2.93 a	33.7 ± 0.89 a
aT + wa	19.2 ± 0.32 b	1.24 ± 0.154 b	0.060 ± 0.0031 ab	21.4 ± 2.05 c	23.3 ± 1.74 b
eT + wa	23.7 ± 1.57 a	1.96 ± 0.124 a	0.053 ± 0.0023 b	37.8 ± 1.31 a	26.2 ± 0.97 b

Note: aT + co, eT + co, aT + wa, and eT + wa indicate 20 °C + controlled irrigation, 30 °C + controlled irrigation, 20 °C + waterlogging, and 30 °C + waterlogging, respectively. The data shown are means of three replicates ± SEM. Different letters indicate significant differences between the four treatments (ANOVA, *p* < 0.05).

## Data Availability

All the data are contained within the article.

## References

[1] Masson-Delmotte V., Zhai P., Pörtner H.-O., Roberts D., Skea J., Shukla P.R., Pirani A., Moufouma-Okia W., Péan C., Pidcoc R., IPCC (Intergovernmental Panel on Climate Change) (2018). Global Warming of 1.5 °C: An IPCC Special Report on the Impacts of Global Warming of 1.5 °C above Pre-Industrial Levels and Related Global Greenhouse Gas Emission Pathways, in the Context of Strengthening the Global Response to the Threat of Climate Change, Sustainable Development, and Efforts to Eradicate Poverty.

[2] Secretariat I.P., Gullino M.L., Albajes R., Al-Jboory I., Angelotti F., Chakraborty S., Garrett K.A., Hurley B.P., Juroszek P., Makkouk K. (2021). Scientific review of the impact of climate change on plant pests. Proceedings of the Secretariat of the International Plant Protection Convention.

[3] Allakhverdiev S.I., Kreslavski V.D., Klimov P.V.V., Los D.A., Carpentier R., Mohanty P. (2008). Heat stress: An overview of molecular responses in photosynthesis. Photosynth. Res..

[4] Zhou R., Wang Q., Jiang F., Cao X., Sun M., Liu M., Wu Z. (2016). Identification of miRNAs and their targets in wild tomato at moderately and acutely elevated temperatures by high-throughput sequencing and degradome analysis. Sci. Rep..

[5] Bailey-Serres J., Fukao T., Gibbs D.J., Holdsworth M.J., Lee S.C., Licausi F., van Dongen J.T. (2012). Making sense of low oxygen sensing. Trends Plant Sci..

[6] Mondal S., Khan M.I.R., Dixit S., Cruz P.C.S., Septiningsih E.M., Ismail A.M. (2020). Growth, productivity and grain quality of AG1 and AG2 QTLs introgression lines under flooding in direct-seeded rice system. Field Crops Res..

[7] Zhao N., Li C., Yan Y., Wang H., Wang L., Jiang J., Chen S., Chen F. (2022). The transcriptional coactivator CmMBF1c is required for waterlogging tolerance in Chrysanthemum morifolium. Hortic. Res..

[8] Sasidharan R., Bailey-Serres J., Ashikari M., Atwell B.J., Colmer T.D., Fagerstedt K., Voesenek L.A. (2017). Community recommendations on terminology and procedures used in flooding and low oxygen stress research. New Phytol..

[9] van Veen H., Akman M., Jamar D.C., Vreugdenhil D., Kooiker M., van Tienderen P., Sasidharan R. (2014). Group VII ethylene response factor diversification and regulation in four species from flood-prone environments. Plant Cell Environ..

[10] Pan J., Sharif R., Xu X., Chen X. (2021). Mechanisms of waterlogging tolerance in plants: Research progress and prospects. Front. Plant Sci..

[11] Kuai J., Liu Z., Wang Y., Meng Y., Chen B., Zhao W., Oosterhuis D.M. (2014). Waterlogging during flowering and boll forming stages affects sucrose metabolism in the leaves subtending the cotton boll and its relationship with boll weight. Plant Sci..

[12] Yan K., Zhao S., Cui M., Han G., Wen P. (2018). Vulnerability of photosynthesis and photosystem I in Jerusalem artichoke (*Helianthus tuberosus* L.) exposed to waterlogging. Plant Physiol. Biochem..

[13] Zhou R., Yu X., Kjær K.H., Rosenqvist E., Ottosen C.O., Wu Z. (2015). Screening and validation of tomato genotypes under heat stress using F_v_/F_m_ to reveal the physiological mechanism of heat tolerance. Environ. Exp. Bot..

[14] Kołton A., Kęska K., Czernicka M. (2020). Selection of tomato and cucumber accessions for waterlogging sensitivity through morpho-physiological assessment at an early vegetative stage. Agronomy.

[15] Yin J., Niu L., Li Y., Song X., Ottosen C.O., Wu Z., Zhou R. (2023). The effects of waterlogging stress on plant morphology, leaf physiology and fruit yield in six tomato genotypes at anthesis stage. Veg. Res..

[16] Zhou R., Niu L., Yin J., Jiang F., Wang Y., Zhao T., Zhu W. (2023). Differences in physiological responses of two tomato genotypes to combined waterlogging and cadmium stresses. Antioxidants.

[17] Lin H.H., Lin K.H., Syu J.Y., Tang S.Y., Lo H.F. (2016). Physiological and proteomic analysis in two wild tomato lines under waterlogging and high temperature stress. J. Plant Biochem. Biotechnol..

[18] Mittler R. (2006). Abiotic stress, the field environment and stress combination. Trends Plant Sci..

[19] Zhou R., Yu X., Song X., Rosenqvist E., Wan H., Ottosen C.O. (2022). Salinity, waterlogging, and elevated [CO_2_] interact to induce complex responses in cultivated and wild tomato. J. Exp. Bot..

[20] Pandey P., Ramegowda V., Senthil-Kumar M. (2015). Shared and unique responses of plants to multiple individual stresses and stress combinations: Physiological and molecular mechanisms. Front. Plant Sci..

[21] Niu L., Jiang F., Yin J., Wang Y., Li Y., Yu X., Zhou R. (2023). ROS-mediated waterlogging memory, induced by priming, mitigates photosynthesis inhibition in tomato under waterlogging stress. Front. Plant Sci..

[22] Barrett-Lennard E.G. (2003). The interaction between waterlogging and salinity in higher plants: Causes, consequences and implications. Plant Soil.

[23] Sasidharan R., Voesenek L.A., Perata P. (2021). Plant performance and food security in a wetter world. New Phytol..

[24] Bradford K.J., Hsiao T.C. (1982). Stomatal behavior and water relations of waterlogged tomato plants. Plant Physiol..

[25] Haldimann P., Feller U.R.S. (2005). Growth at moderately elevated temperature alters the physiological response of the photosynthetic apparatus to heat stress in pea (*Pisum sativum* L.) leaves. Plant Cell Environ..

[26] Yuan M., Cai C., Wang X., Li G., Wu G., Wang J., Sun Y. (2021). Warm air temperatures increase photosynthetic acclimation to elevated CO_2_ concentrations in rice under field conditions. Field Crops Res..

[27] Hikosaka K., Ishikawa K., Borjigidai A., Muller O., Onoda Y. (2006). Temperature acclimation of photosynthesis: Mechanisms involved in the changes in temperature dependence of photosynthetic rate. J. Exp. Bot..

[28] Berry J., Bjorkman O. (1980). Photosynthetic response and adaptation to temperature in higher plants. Annu. Rev. Plant Physiol..

[29] Sharkey T.D., Bernacchi C.J., Farquhar G.D., Singsaas E.L. (2007). Fitting photosynthetic carbon dioxide response curves for C3 leaves. Plant Cell Environ..

